# Prediction of perioperative outcome after hepatic resection for pediatric patients

**DOI:** 10.1186/s12876-019-1109-7

**Published:** 2019-11-27

**Authors:** Jianxia Liu, Yunfei Zhang, Hai Zhu, Lin Qiu, Chunbao Guo

**Affiliations:** 1 Department of Anesthesiology, Children’s Hospital, Chongqing Medical University, Chongqing, People’s Republic of China; 2 Department of Pediatric General Surgery, Children’s Hospital of Chongqing Medical University, 136 Zhongshan 2nd Rd., Chongqing, 400014 People’s Republic of China; 3 Ministry of Education Key Laboratory of Child Development and Disorders, Children’s Hospital, Chongqing Medical University, Chongqing, People’s Republic of China; 4 Department of Pediatric General Surgery and Liver Transplantation, Children’s Hospital of Chongqing Medical University, 136 Zhongshan 2nd Rd., Chongqing, 400014 People’s Republic of China

**Keywords:** Hepatectomy, Pringle maneuver, Prediction, Estimated blood loss

## Abstract

**Background:**

Hepatic resection is associated with significant risk of morbidity and mortality. Optimising the surgical techniques and perioperative management may improve in operative morbidity and mortality. However, perioperative variables involved in the improvement for postoperative outcomes in pediatric hepatectomy have not been defined.

**Methods:**

We retrospectively reviewed 156 consecutive pediatric patients who underwent hepatectomy at our center (an academic tertiary care hospital) between 2006 and 2016. Baseline demographic variables, intraoperative variables, complications, and hospital stay were explored. The patients were further investigated using univariate and multivariate analysis for the factors involved in the postoperative outcomes.

**Results:**

Of the conditions requiring resections, malignant and benign liver diseases accounted for 47.4% (74/156) and 52.6% (82/156), respectively. The overall hospital mortality was 1.9% (3/156) and the overall postoperative complication rate was 44.2% (69/156). Anatomical resections were performed in 128 patients (82.1%), including 14(9.0%) extended hepatectomies. Eighty percent of patients had three or more segments resected. The median operative time was 167.7 (65–600) minutes and median estimated blood loss was 320.1(10–1600) mL. On multivariate analysis, the estimated blood loss (EBL) (mL) (OR, 2.19; 95CI, 1.18–3.13; *p* = 0.016), extent of hepatectomy (OR, 1.81; 95CI, 1.06–2.69; *p* = 0.001) and pringle maneuver (OR, 1.38; 95CI, 1.02–1.88; *p* = 0.038) were the independent predictors of postoperative complications.

**Conclusions:**

Extent of hepatectomy and estimated blood loss are largely responsible for the perioperative complications. With the surgical devices and management amelioration, like pringle maneuver, the treatment planning may be optimize in pediatric liver resection.

## Background

Hepatectomy is one of the major abdominal procedures, with a notable change in their natural history. Currently hepatectomy is a increasingly performed in the treatment of various hepatobiliary diseases, with substantial improvement in long-term survival as a result of recent advances in surgical techniques and perioperative care [1–5].

Over the past few years, the hepatobiliary surgery have resulted in good perioperative results in several centres, with operative mortality rates lower than 5% [6, 7]. As a result, hepatectomies are performed with greater frequency with the feasibility of segment-oriented resections and indications for liver resection have broadened in patients with a normal liver [8]. When major hepatic resection is required, especially for patients with chronic liver disease, the hepatic resection is still challenging. In addition, the procedure is still burdened by some postoperative complications and mortality. Thus, the hepatic resection still evolved with the progress of surgical techniques.

To further improve the clinical outcomes, it is important to identify the prediction of hepatectomy complications, which may allow heightened vigilance, and corresponding intervention [9]. A few recent studies have specifically evaluated the perioperative outcome of hepatectomy but very little pediatric patients involved if any [10, 11]. Detailed information about incidence and risk factors for pediatric hepatectomy has not been available [12]. In addition, whether events like massive blood loss, inflammatory insults during liver resection are potentially related to some pediatric conditions needs to be elucidated.

In this study, we reviewed a consecutive series of pediatric patients undergoing hepatic resection in our institute, and we sought to define the risk factors associated with perioperative morbidity and mortality, which would hopefully help to a better clinical practice and good patient outcome.

## Methods

This was an retrospective observational study for a series of patients underwent hepatectomy for benign or malignant hepatobiliary diseases, from July 1st, 2006 to September 1st, 2016 at the Department of General Surgery and Transplantation, Chongqing Medical University, China. Indications for resection are shown in Table 1. The patients requiring primary hepatectomy were considered eligible for entry into the study upon meeting the following inclusion criteria: no severe sepsis; no steroid or immunosuppressive medication administration. Patients who underwent a liver biopsy only were excluded. All the hepatectomies information including preoperative and intraoperative parameters were stored in the medical records, like demographic data, the clinical details of all the cases, preexisting comorbidities, etc. Following institutional review board approval, we retrospectively reviewed the electronic medical records of all this patients by investigators who had undergone our specific training. Patient background demographics included age, sex, previous major abdominal surgery, and comorbidities. Preoperative variables analyzed included diagnoses, American Society of Anesthesiology classification (ASA), and laboratory values (bilirubin, albumin, creatinine, platelet count). Intraoperative data were obtained from the operative note and the anesthesia record and included duration of operation and anesthesia, operating time, portal triad clamp time (pringle time), estimated blood loss (EBL), intraoperative blood transfusion, etc. The postoperative variables analyzed included nasogastric tube stay, parenteral nutrition duration, complications (any), hospital length of stay (LOS), intensive care unit (ICU) admission, mortality and necessity for re-operation.

**Table 1:** Baseline demographics and clinical characteristics for eligible cohort (*n* = 156)

Variables	
Male: female	91:65
Age (yrs)	4.4 (range: 1 month–11.7 years)
Weight (kg)	13.8 (range: 2.9–58.1 kg)
Co-morbidities, n (%)	
Malnutrition	36 (23.1%)
Portal hypertension	9 (5.8%)
Hypersplenism	8 (5.1%)
Hypoproteinemia	31 (19.9%)
Anaemia	18 (11.5%)
Chemotherapy within 30 days of operation	22 (14.1%)
Preoperative portal vein embolization	8 (5.1%)

### Preoperative assessment

The formal preoperative evaluation included a thorough preoperative history and physical examination, liver function tests, tumor markers measurement, routine chest x-ray film, routine cardiorespiratory evaluations, abdominal ultrasound, abdominal computed tomography (CT) scan and/or magnetic resonance (MR), in all cases. According to the Couinaud’s classification, anatomic resectability was evaluated in which the lesion(s) are completely anatomically removed on the basis of tolerable functional reserves, including segmentectomy, sectionectomy, hemihepatectomy, and extended hemihepatectomy. Nonanatomic partial resection was defined as a limited resection. According to the preference and experience of the attending surgeon, the surgical team determined the type of procedure for all the patients. To decrease the risk of postoperative liver failure, compensatory hypertrophy of the estimated remnant liver were induced through portal vein embolization (PVE) 3 to 6 weeks before liver resection when the volume of the future remnant liver was 25% or less in healthy livers and 40% or less in chronic hepatitis or cirrhosis livers.

### Surgical procedure

Patients undergoing operation were set in the Trendelenburg position and explored through right or bilateral subcostal incision. All patients underwent a complete abdominal exploration and intraoperative ultrasound. To prevent venous hemorrhage, the central venous pressure was maintained below 5 cm H_2_O in all hepatectomies.

Liver resection was performed with an ultrasonic dissector (Ethicon Inc., Somerville, NJ). Major portal triads and hepatic veins were carefully exposed with the hook spatula Vessels greater than 2 mm in diameter were ligated or sutured with nonabsorbable sutures. Vessels less than 2 mm in diameter were coagulated with the bipolar cautery. Hemihepatic vascular occlusion, selective portal triad clamping and pringle maneuver were arbitrarily chosen alone or in combination with 15 min of occlusion alternated with 5 min of reperfusion.

The segmental resection was performed anatomically for one or more segments: five segments, extended hepatectomy; four segments, lobectomy (right hepatectomy); three segments, left hepatectomy (lobectomy), central hepatectomy; two segments, left lateral segmentectomy, right anterior or posterior sectorectomy; one segment, a wedge resection. Bilobar resections were considered as resection of segments from both the left and right hemilivers. An enucleation was defined zero segments resected. An abdominal drain was given routinely after hepatic resection.

### Postoperative management

Following operation, patients were not routinely admitted to the intensive care unit. A abdominal drain tube was usually placed during the operation, and routinely removed 3 days after hepatectomy. When the perioperative hemoglobin value fell below 8 g/L, blood transfusions were carried out. Abnormality of coagulation parameters were the indication for Fresh frozen plasma (FFP) transfusions, and intravenous albumin was given for patients with hypoalbuminemia. A broad-spectrum antibiotic was given routinely for 3 d. Oral feeding was restored when the bowel sounds returned and postoperative parenteral nutrition was administered when necessory.

### Postoperative outcomes

Operative mortality was defined as any death resulting from the operation during the postoperative hospital stay. Postoperative morbidity included all the complications derived from the liver resection or associated procedure. Liver-specific complications commonly encountered after major liver procedures were assessed, including liver failure, ascites, bile leakage. Other complications happened were also recorded, like wound infection or dehiscence, intra-abdominal infection, postoperative hemorrhage, small bowel occlusion, subphrenic abscess, bronchopneumonia, and so on.

### Statistical analysis

Preoperative, operative and postoperative variables were expressed as the means ± SD for continuous data and as numbers with percentages for categorical data. The association of variables with postoperative complications were evaluated using the chi-square test or Fisher exact test for categorical variables and the Student t-tests or rank sum test for continuous variables, when appropriate, respectively. Variables with statistical significance at the 0.10 level in univariable analysis were considered for multivariable analysis to determine the predictive value of the risk factors, which was performed by means of logistic regression. All statistical analyses were performed using the SPSS statistical package (version 17.0, SPSS Inc., Chicago, IL), and statistical significance was accepted at a two-tailed *P* value < 0.05.

## Results

### Demographics

During the study period, one hundred eighty seven consecutive patients undergoing hepatectomy were considered for eligibility; 31 cases were excluded due to incomplete information, and 156 cases were included in the final analysis. Among the 156 patients, there were 91 male (58.3%) and 65 female (31.7%) with the mean age of 4.41 years (range 1 month-11.7 years) years (Table 1). The indication for hepatectomy was benign disease in 82(52.6%) patients and malignant disease in 74 patients (47.4%), hepatoblastoma being the most common disease, which were summarized in Table 2. Fifty-two patients (33.3%) had at least one comorbidity, most commonly malnutrition (23.1), portal hypertension (5.8%) and hypersplenism (5.1%). Preoperative laboratory abnormalities were common, including, hypoproteinemia (19.9%), and anaemia (11.5%), and so on. Some 22 patients (14.1%) had undergone preoperative chemotherapy. Preoperative portal vein embolization was performed in 8 (5.1%) patients due to anticipated insufficient remnant liver.

**Table 2:** Indications for the pediatric hepaticresection for 156 cases

Diagnoses	n (%)
Benign	82 (52.6%)
Hemangioma	31 (19.9%)
Hamartoma	8 (5.1%)
Focal nodular hyperplasia	5 (3.2%)
Adenoma	1 (0.64%)
Hepatic cyst	13 (8.3%)
Hydatid disease	3 (1.9%)
Hepatic laceration	14 (9.0%)
Other	7 (4.5%)
Malignant	74 (47.4%)
Hepatoblastoma	58 (37.2%)
Hepatocellular carcinoma (HCC)	5 (3.2%)
Sarcoma	3 (1.9%)
Metastatic disease	2 (1.3%)
Other	6 (3.8%)

### Intraoperative variables

The intraoperative variables for all patients are summarized in Table 3. The average operative time was 167.7 (range 65–600) min and the average estimated blood loss (EBL) was 320.1 (range 10–1600) ml. Only 29 patients (18.6%) were classed as low American Society of Anesthesiologists fitness grade II. One hundred forty four patients (92.3%) required transfusion of blood products during or after operation. The median intraoperative crystalloid volume administered among all patients was 899.1(range, 120–6400) mL, which were varied based on patient weight and operative duration. One hundred and one patients (64.7%) underwent anatomically based major hepatectomy, which were predominantly right or left hemihepatectomy (59, 37.8%), followed with central hepatectomy in eight (7, 4.5%), extended right or left hemihepatectomy (14, 9.0%), left lateral segmentectomy (12, 7.7%), or right anterior or posterior sectorectomy (9, 5.8%). Twenty-seven patients (27, 17.3%) underwent a segmental resection. Nonanatomical wedge resections or enucleations were performed in 28 patients (17.9%). Twenty (12.8%) patients underwent major hepatectomy with at least one additional major procedure, including resection and reconstruction of the vena cava (3, 1.9%); portal vein resections and reconstructions (5, 3.2%); resection and reconstruction of the hepatic artery (12, 7.7%). There were 80 patients (51.3%) underwent resection of three or more segments and the average number of hepatic segments resected was 2.8 ± 0.6 (range 0–6) among all the patients.

**Table 3:** Intraoperative characteristics for the pediatric hepaticresection (*n* = 156)

Variables	
ASA fitness grade, n (%)	
II	29 (18.6%)
III	119 (76.3%)
IV	8 (5.1%)
Operative time (mins), median (range)	167.7 (65–600)
Estimated blood loss (ml), median (range)	320.1 (10–1600)
Extent of liver resection, n (%)	
Extended right or left hemihepatectomy	14 (9.0%)
Right hemihepatectomy	36 (23.1%)
Left hemihepatectomy	23 (14.7%)
Central hepatectomy	7 (4.5%)
Left lateral segmentectomy	12 (7.7%)
Right anterior or posterior sectorectomy	9 (5.8%)
Segmental resection	27 (17.3%)
Wedge resections	16 (10.3%)
Enucleations	12 (7.7%)
Additional major procedure, n (%)	
Resection and reconstruction of the vena cava	3 (1.9%)
Portal vein resections and reconstructions	5 (3.2%)
Resection and reconstruction of the hepatic artery	12 (7.7%)
Over three segments resection, n (%)	80 (51.3%)
Transfused patients, n (%)	144 (92.3%)
Red cell transfusion (units), median (range)	1.81 (0–11.75)
FFP transfusion (ml), median (range)	163.65 (0–1500)
Nadir Hb concentration (g/dL), median (range)	86.4 (72–144)
Crystalloid (mL), median (range)	899.1 (120–6400)

### Perioperative outcomes

According to established criteria, the perioperative outcomes were summarized in Table 4. The mean hospitalization time was 15.3(range, 6–62) days, and 111 patients (71.2%) required ICU admission. The total perioperative complication rate was 69.2% (*n* = 108), with 21.8% of patients (*n* = 34) experiencing multiple complications. The most common complication was infection, occurred in 39 patients (25.0%), following with intestinal occlusion (*n* = 22, 14.1%), ascites (*n* = 21, 13.5%), biliary leakage (*n* = 18, 11.5%), liver failure (*n* = 4, 2.6%), intra-abdominal abscess (*n* = 9, 5.8%), intra-abdominal bleeding (*n* = 7, 4.5%), and wound dehiscence (*n* = 3, 3.7%). Reoperations were needed for nine patients (5.8%) because of postoperative intra-abdominal bleeding in four, biliary leakage in one, wound dehiscence in three and postoperative small bowel obstruction in one patient. Three patients died in the postoperative period, with overall in-hospital mortality was 1.9% (3/156). One patient died from multisystem organ failure following extended right hemihepatectomy. One died of severe sepsis. The last dead one resulted from massive intra-abdominal bleeding in postoperative day 12.

**Table 4:** Perioperative outcomes for the pediatric patients undergoing hepatic resection

Hospital stay (days), median (range)	15.3 (6–62)
Patients requiring ICU admission, n (%)	111 (71.2%)
Total complications	108
Patients experiencing>1complication, n (%)	69 (44.2%)
Patients experiencing> 2 complications, n (%)	34 (21.8%)
Infections, n (%)	39 (25.0%)
Abdominal bleeding/hematoma, n (%)	7 (4.5%)
Transient liver failure, n (%)	4 (2.6%)
Bile leakage, n (%)	18 (11.5%)
Perihepatic fluid collection, n (%)	11 (7.1%)
Intra-abdominal abscess, n (%)	9 (5.8%)
Ascites, n (%)	21 (13.5%)
Wound dehiscence, n (%)	3 (1.9%)
Intestinal occlusion, n (%)	22 (14.1%)
Others, n (%)	7 (4.5%)
Reoperations, n (%)	9 (5.8%)
Mortality, n (%)	3 (1.9%)
Liver failure, n (%)	1 (0.64%)
Digestive bleeding, n (%)	1 (0.64%)
Sepsis, n (%)	1 (0.64%)

### Factors associated with perioperative complications

We next explored the associations of various pre- and intra-operative variables and perioperative complications (Table 5). In univariable analysis, there were significant shorter operation duration (*p* < 0.001), lower ASA (*p* = 0.001), and less intraoperative blood loss (*p* = 0.003) for the 87 patients without complications compared with the 69 patients with complications. The pringle maneuver was performed more in the 69 patients with complications (*p* = 0.026) compared with the patients without. The number of hepatic segments resection had a significant impact on perioperative complications, with 14.5% (11/76) for patients who underwent a resection of zero or two segments compared with 72.3% (58/80) in patients who underwent resection of over 3 segments (*p* < 0.001).

**Table 5:** Univariate analysis of factors associated with perioperative complications

Characteristics	With complications (69)	No complications (87)	*p* Values
Male: female	40:29	51:36	NS
Age (ys) mean ± SD	4.4 ± 1.2	4.5 ± 1.3	NS
weight (g) mean ± SD	13.4 ± 4.1	14.2 ± 4.5	NS
Preoperative Alb (g/L)	41.6 ± 3.5	40.8 ± 3.1	NS
Preoperative T bil(mmol/L)	11.3 ± 5.2	11.9 ± 4.8	NS
Preoperative ALT (U/L), median (range)	69 (6.4–6511)	60 (10.3–2888)	NS
Preoperative AST (U/L), median (range)	72 (16.7–7221)	67 (20–3486)	NS
Preoperative PT (s)	11.6 ± 1.6	12.2 ± 1.5	NS
Preoperative Hemoglobin (g/L)	95.2 ± 11.4	94.6 ± 12.1	NS
ASA, n (%)			
II	5 (7.2)	24 (27.6)	0.001
III	56 (81.2)	63 (72.4)	0.14
IV	8 (11.6)	0	0.001
Operation duration (min), mean ± SD	179.2 ± 61.8	158.2 ± 59.7	< 0.001
Pringle maneuver, n (%)	21 (30.4)	12 (13.8)	0.026
Extent of hepatectomy, n (%)			
≥ 3 Segments	58 (84.1)	22 (25.3)	
< 3 Segments	11 (15.9)	65 (74.7)	< 0.001
Blood transfused (mL)			0.050
EBL (mL), mean ± SD	338.1 ± 91.6	306.8 ± 98.4	0.003
Urine output (mL), mean ± SD	345.2 ± 103.9	329.5 ± 99.7	0.039
Diagnosis, n (%)			
Benign	19 (27.5)	63 (72.4)	
Malignant	50 (72.5)	24 (27.6)	< 0.001
Crystalloid (mL), mean ± SD	924.6 ± 214.5	875.2 ± 203.9	0.057
First pass of stool, mean ± SD	2.62 ± 1.4	3.12 ± 1.4	0.041
Parenteral nutrient (d), mean ± SD	3.18 ± 1.65	3.06 ± 1.47	0.004

Multivariate analysis revealed that EBL (OR, 2.19; 95CI, 1.18–3.13; *p* = 0.016), number of segments resected (OR, 1.81; 95CI, 1.06–2.69; *p* = 0.001), and use of pringle maneuver (OR, 1.38; 95CI, 1.02–1.88; *p* = 0.038) were associated with an increased risk of developing perioperative complications (Table 6).

**Table 6:** Multivariable analyses of the factors with perioperative complications

Variables	Odds ratio (95% CI)	*p* values
EBL (mL)	2.19 (1.18–3.13)	0.016
Extent of hepatectomy	1.81 (1.06–2.69)	0.001
Pringle maneuver	1.38 (1.02–1.88)	0.038

## Discussion

Hepatectomy is a heavy technique and devices dependency operation, with high postoperative complications, although the mortality is generally very infrequent. This retrospective observational study for the first time detected the independent risk for complications following hepatectomy using multivariate logistic regression analysis, which could be evaluated by operative magnitude, like operative time, number of segments resection, estimated blood loss, and total units of blood transfused measurement. Furthermore, a pringle maneuver was significantly associated with perioperative complications.

Previous research detected that massive blood loss correlated with morbidity and mortality after hepatic resection [13]. Although there was no similar report from pediatrics, the current data illustrated the importance of EBL and the associated blood transfusion in determining outcome of hepatic resection. The blood transfusion might cause an immunosuppressive state, which should contribute to postoperative infections and other associated events [14, 15]. The increase in complex resections may explain the greater operating time, EBL and ICU admissions. A higher number of extended hepatectomies had a much more profound impact for EBL and operative complications [16]. A major hepatic resection (≥3 segments) was more performed for the malignant diseases, which were associated with significantly increasing EBL, transfusion requirements, and postoperative complications.

Today, in order to reduce blood loss and shorten the operative time, various techniques new devices have been developed [17–19]. The surgical techniques included intermittent inflow occlusion, low central venous pressure, hepatic vascular exclusion (THVE), harmonic scalpel and coagulating device (bipolar cautery), etc. The low amount of blood loss and rate of transfusion were achieved even when undergoing a major liver resection [20]. From 2006, we resected hepatic parenchyma using the cavitron ultrasonic surgical aspirator (CUSA) and a saline irrigation system for dissecting the soft liver parenchyma. Vessels that were firmer than the liver parenchyma could be easily exposed for coagulates or ligation. But in our pediatric series, under this comprehensive measures, the median blood loss was 320.1(10–1600) mL and the transfusion rate was 92.3%, which was still high compared with data from previous reports from adult, suggesting the particularity and difficulty for pediatric hepatectomy.

Although pringle maneuver is effective in reducing blood loss, long time for inflow occlusion can bring about multiple remote organ injuries, like gut barrier dysfunction and other complications [21, 22]. For the pringle maneuver performance, the hepatic veins dissection is extremely hazardous, so need special training [21]. To our knowledge, few studies have directly probed into the impact of pringle maneuver on clinical complications following pediatric hepatic resection. In the current cohort patients, 43 patients were adopted pringle maneuver for extended hepatic resection, which were taken a high percentage compared with other reports. Due to the higher incidence of complications in those experienced more numbers of segment resection, there was also high in the need for pringle maneuver. Our current data showed that the pringle maneuver was associated with comparatively more complications, which are in accordance with previous report [23]. This is likely due to the fact that those who were on higher operative magnitude with higher blood loss that was more predisposed to pringle maneuver. Although no studies have looked directly at the impact of pringle maneuver on postoperative complications, few have investigated the effects of pringle maneuver on gut barrier dysfunction. Our study was specifically aimed at exploring the potential risk of morbidity for pediatric hepatic resection, suggests that the pringle maneuver can be a risk factor for morbidity for pediatric hepatic resection. Of cause, this finding should be taken in context of other known risk factors for hepatic resection in pediatric patients. Moreover, many surgeons have performed hepatectomy successfully in absence of pringle maneuver, suggesting that it is not obligatory for liver resections [24, 25]. It should be noted that the central venous pressure (CVP) fluctuations highly influence the effectiveness of the pringle maneuver. Liver resections with a high CVP apparently promote hemorrhage and a low CVP may also give rise to hemorrhage due to undetected incompletely sutured vessels. In our institute, the CVP was strictly controlled within the range of 3–6 mmHg though anesthetic management, which should ensure without tissue hypoperfusion and air embolism [26]. In the current cohort, there was no air embolism event.

This present study has several limitations. Primarily, a major limitation for the current data was the retrospective analysis over a long study interval, in which detailed data were unavailable in certain patients. Also, there might be many practice changes for care practices, including the procedure and the anesthetic management among the patients. In practice, there might be an inclination to adopt the pringle maneuver in the patients prone to multiple segments resection with more blood loss, which should be associated with long procedure time and tend to transfer into ICU care. Given the retrospective design, and relatively heterogeneous samples, the current difference in the pringle maneuver must be interpreted with caution. A power prospective analysis including large sample size may better clarify the surgical outcomes of hepatectomy.

## Conclusion

The present study documented encouraging predictor information for pediatric hepatectomy, which may warrant further attention in studies on optimizing intraoperative management, such as pringle maneuver usage and blood loss control. Further studies evaluating its impact on the decision making process should be taken on larger data sets to facilitate a better quality of care.

## Data Availability

The datasets during and/or analyzed during the current study are available from the corresponding author on reasonable request.

## Abbreviations

AEs: Adverse events
ASBO: Adhesive small bowel obstruction
CI: Confidence interval
CRP: C-reactive protein
CT: Computed tomography
GI: Gastrointestinal
IQR: Interquartile range
LOS: Hospital length of stay
NGT: Nasogastric tube
OR: Odds ratio
POI: Postoperative ileus
RR: Risk ratio
